# Breaking Barriers in Interdisciplinary Research: The Case for a Unified Approach in Sports Science and Public Health

**DOI:** 10.3390/sports13030082

**Published:** 2025-03-10

**Authors:** Andrew M. Lane, Richard B. Kreider

**Affiliations:** 1 School of Psychology, University of Wolverhampton, Wolverhampton WV1 1LY, UK; 2 Exercise and Sport Nutrition Laboratory, Human Clinical Research Facility, Department of Kinesiology and Sports Management, Texas A&M University, College Station, TX 77843-4253, USA

## 1. Introduction

There has been a long-standing call in the sports and exercise sciences for researchers to adopt an interdisciplinary approach [1,2]. True interdisciplinarity is a challenging yet essential pursuit, particularly in fields that aim to improve public health, such as sports science, exercise, and physical activity [3]. While interdisciplinary collaboration is widely supported, its practical implementation often encounters significant barriers [1,2,4,5]. One of the most substantial hurdles is the distinctiveness of the disciplines involved, each with its own terminology, methodologies, foundational knowledge, and cultural influences. Although valuable in their specific domains, these differences can create walls that impede effective collaboration. The divide between disciplines and areas of application, especially in the context of sports, exercise, and physical activity, has created larger barriers than necessary. These areas should be seen as applications of various disciplines rather than separate entities. When viewed through an interdisciplinary lens, sports science has the potential to raise public health outcomes by bringing together the strengths of disciplines commonly associated with sports and exercise science, such as psychology, physiology, and biomechanics, and also academic subjects uncommonly associated with the discipline, such as physics and chemistry. The true power of interdisciplinary research lies not in merging disciplines into one but in fostering the kind of enthusiasm and interaction that allows each discipline to inform and enrich the others.

## 2. Bridging the Gap

At the heart of the challenge is the distinction between a discipline and an area of application. Disciplines like psychology, physiology, and chemistry each have well-established bodies of knowledge, methods, and systems of inquiry that help researchers make sense of complex phenomena. In contrast, sports, exercise, and physical activity are often treated as applications of these disciplines, focusing more on practical outcomes than theoretical exploration. The divide between these categories, however, has created unnecessary silos that limit the ability to leverage the full potential of interdisciplinary collaboration. While disciplines provide the depth of knowledge required to understand specific aspects of human performance and health, areas of application like sports and physical activity are where this knowledge can be put to use for societal benefit. The boundary between them is not as rigid as it appears, and the artificial separation between research and practice can be detrimental to both the advancement of science and the improvement of public health.

The interdisciplinary nature of sports science is vital for promoting better public health. Sports and physical activity have a direct impact on the health and well-being of individuals and populations, influencing everything from cardiovascular health to mental well-being. However, to fully understand and harness the potential of these fields, insights from diverse disciplines are required. Psychology, for instance, plays a crucial role in understanding motivation, behavior change, and the psychological benefits of physical activity. The mental health benefits of exercise are well-documented, with research showing how physical activity can reduce symptoms of depression and anxiety, boost mood, and improve cognitive function. Psychological theories of behavior change, such as the Theory of Planned Behavior or Self-Determination Theory, provide valuable frameworks for designing interventions that encourage lifelong physical activity.

Physiology, on the other hand, offers the foundational understanding of how the human body responds to exercise. The physiological processes of muscle adaptation, energy production, and cardiovascular health are central to understanding how different types of exercise can improve physical fitness and reduce the risk of chronic diseases. Without this knowledge, recommendations for physical activity would lack scientific rigor, making it difficult to promote effective health interventions. In this context, the collaboration between psychology and physiology becomes particularly important, as understanding both the psychological drivers of behavior and the physiological responses to exercise can lead to more effective, tailored interventions that encourage healthier lifestyles.

The disciplines of physics and chemistry also have vital roles to play in the broader field of sports science. Biomechanics, a branch of physics, helps explain how the body moves, how forces are generated and absorbed during physical activity, and how to optimize performance while reducing the risk of injury. The application of physical principles in sports science is key to understanding not only athletic performance but also the mechanics of rehabilitation and recovery. Similarly, chemistry offers insights into metabolism, the biochemical processes that fuel our bodies during exercise. The interaction of nutrients, hormones, and enzymes during physical activity and dietary influences is essential for understanding how to optimize performance, prevent fatigue, and recover effectively. Moreover, the prevention and rehabilitation of injuries affects the ability of individuals to engage in physical activity in order to improve health and/or performance. These insights from physics and chemistry complement those from psychology, physiology, and rehabilitation sciences, thereby creating a more holistic understanding of the body’s response to exercise.

These are a selection of the different disciplines available, and for these disciplines to work effectively together, enthusiasm and open communication are essential. Too often, researchers in one discipline may view the methods or assumptions of another as irrelevant or impractical. However, interdisciplinary research is most successful when there is a shared enthusiasm for collaboration and a willingness to learn from one another. Each discipline brings a unique perspective that can enhance the others, and the synthesis of these perspectives can lead to breakthroughs that would not be possible within the confines of a single discipline. For instance, psychologists studying motivation can collaborate with physiologists to design exercise programs that not only meet the physical demands of the body but also engage participants at a psychological level, fostering long-term adherence to healthy behaviors.

One of the key barriers to true interdisciplinarity is the language and methodology of different disciplines. Each field has its own set of tools, techniques, and terms, which can make communication challenging. This divide is often further entrenched by the training and education systems that emphasize specialization over generalist thinking. However, the complexity of public health issues, particularly those related to physical activity, requires solutions that draw on knowledge from a range of disciplines. To overcome these challenges, researchers must work to develop a shared language that allows them to communicate across disciplines effectively. Additionally, institutions must foster an environment where interdisciplinary collaboration is not only encouraged but actively supported through funding, training, and the creation of collaborative research spaces.

Sports science, exercise, and physical activity are not isolated fields of study but should be seen as areas of application where knowledge from disciplines like psychology, physiology, physics, and chemistry can be applied to improve public health. The divide between disciplines and areas of application has created unnecessary barriers that limit the potential for meaningful collaboration. True interdisciplinarity requires enthusiasm and openness from all parties involved and an understanding that each discipline brings valuable insights to the table. By breaking down these barriers and fostering interdisciplinary collaboration, we can enhance the effectiveness of interventions aimed at improving health outcomes and creating a more integrated approach to the challenges of public health.

The relationship between academic disciplines and their areas of application is a dynamic and evolving one, as illustrated in the sports science and public health research featured in the Special Issue of the journal *Sports*. We set out to encourage interdisciplinary excellence. These articles showcase how sports science integrates with real-world applications in health, performance, and wellness, often straddling multiple disciplines like psychology, nutrition, physiology, and biomechanics. For example, King et al. (Contribution 1) explore the intersection of injury management and sports science in amateur female soccer, emphasizing how injury data can inform future training and recovery strategies. This highlights the practical application of biomechanics and physiotherapy within sports science, as well as the integration of health data into policy and practice in amateur sports settings. The study emphasizes how theoretical knowledge of injury prevention must translate into actionable strategies for athletes, bridging the gap between research and field application.

Similarly, the research by Magalhães et al. (Contribution 2) on the effects of training intensity distribution in recreational cyclists reveals the interplay between exercise physiology and sports performance. The authors link theoretical models of endurance training to measurable improvements in athletic performance. Their work provides a direct application of training theory to practical fitness programs for everyday athletes, demonstrating how academic discipline in exercise science informs public health recommendations for recreational and professional athletes alike.

Rohlfs et al. (Contribution 3) examine the psychometric characteristics of the Brazil Mood Scale in athletes, showing the link between sports psychology and practical application in mood regulation and performance. The study underscores the importance of psychological assessments in sports science, applying theories of mood and emotion regulation to real-world training environments. These tools, developed within academic psychology, offer direct benefits to athletes and coaches by providing insights into how emotional states affect performance.

Pradas de la Fuente et al. (Contribution 4) focus on technical–tactical actions in high-level table tennis, emphasizing the application of motor control theory and tactical analysis in sports. This research translates academic principles in movement science into tactical strategies that can improve performance outcomes in elite sports, showing how discipline-specific knowledge in areas like motor learning and cognitive psychology impacts competitive sports.

These examples reflect a broader trend where academic research in sports science serves as the foundation for practical interventions, whether for injury prevention, performance enhancement, or psychological well-being. They illustrate how theoretical frameworks are continually tested and refined through applied research, with direct implications for sports practitioners, athletes, and health professionals.

The application of academic discipline to sports science not only advances athletic performance but also contributes to public health, especially as sports and exercise are increasingly recognized for their role in disease prevention and overall well-being. Researchers like Magalhães et al. (Contribution 2) and Merlo et al. (Contribution 5) demonstrate how applied exercise science improves health outcomes by informing public health initiatives aimed at reducing chronic disease through physical activity, emphasizing how academic insights are crucial for societal health challenges. Tryfonos and associates (Contribution 6) reported that L-citruline supplementation (6 g/d for six days) did not enhance blood pressure or arterial stiffness at rest or in response to low-intensity isometric knee extension exercise. Lachbaum et al. (Contribution 7) described the role of pre-event self-efficacy and sports performance. Finally, the role of exercise and nutrition in the management of a runner with type 1 diabetes was articulated by Schroeder and coworkers (Contribution 8). Vlachopoulos et al. (Contribution 9) reported that participants with obesity reported higher negative mood scores than those who were underweight or normal weight using a Greek translation of the Brunel Mood Scale (BRUMS-Greek). Finally, de Souza et al. (Contribution 10) described how physical inactivity during the COVID-19 pandemic due to mandated lockdowns led to an increase in body weight and a worsening of glycemic control. Conversely, individuals with type I and type II diabetes mellitus who maintained or increased physical activity levels had better glucose control and lower hemoglobin A1c levels.

In summary, the integration of academic disciplines such as psychology, physiology, and biomechanics with applied sports science is essential for advancing both performance outcomes and public health. These connections between theory and practice ensure that research has meaningful, real-world applications in improving health and performance across various sectors of society. The 10th-anniversary Special Issue of *Sports* provides a unique opportunity to showcase how interdisciplinary physical activity and sports-related research approaches can affect physical activity, sports, and public health. Moving forward, we encourage practitioners and scholars to work together to develop public health and performance enhancement teams to address transdisciplinary efforts to determine how physical activity and sports can improve performance, psychological well-being, public health, and society.
